# Supplementary material for: Understanding the diabetes self-care behaviour in rural areas: Perspective of patients with type 2 diabetes mellitus and healthcare professionals
Source: PLoS One. 2024 Feb 8;19(2):e0297132. doi: 10.1371/journal.pone.0297132 (PMC10852243; doi:10.1371/journal.pone.0297132)
Supplement: S1 Appendix — (PDF) [file pone.0297132.s001.pdf]

S1 appendix:
Consolidated criteria for reporting qualitative studies (COREQ): 32-item checklist.
Developed by:
Tong A, Sainsbury P, Craig J. Consolidated criteria for reporting qualitative research (COREQ): a 32-item checklist for interviews and focus groups. International Journal for Quality in Health Care. 2007. Volume 19, Number 6: pp. 349 – 357

No. Item	Guide questions/description	Reported on Page #
**Domain 1: Research team and reflexivity**		
Personal Characteristics		
1. Inter viewer/facilitator	Which author/s conducted the interview or focus group?	**Page 7**
2. Credentials	What were the researcher's credentials? E.g. PhD, MD	**Page 7**
3. Occupation	What was their occupation at the time of the study?	**Page 7**
4. Gender	Was the researcher male or female?	**Page 7**
5. Experience and training	What experience or training did the researcher have?	**Page 7**
Relationship with participants		
6. Relationship established	Was a relationship established prior to study commencement?	**Yes/ Page 6-7**
7. Participant knowledge of the interviewer	What did the participants know about the researcher? e.g. personal goals, reasons for doing the research	**Page 6-7**
8.Interviewer characteristics	What characteristics were reported about the inter viewer/facilitator? e.g. Bias, assumptions, reasons and interests in the research topic	**Page 6-7**

**Domain 2: study design**		
Theoretical framework		
9. Methodological orientation and Theory	What methodological orientation was stated to underpin the study? e.g. grounded theory, discourse analysis, ethnography, phenomenology, content analysis	**Page 5-7**
Participant selection		
10. Sampling	How were participants selected? e.g. purposive, convenience, consecutive, snowball	**Page 6-7**
11. Method of approach	How were participants approached? e.g. face-to-face, telephone, mail, email	**Page 6-7**
12. Sample size	How many participants were in the study?	**Page 6-7**
13. Non-participation	How many people refused to participate or dropped out? Reasons?	**None**
Setting		
14. Setting of data collection	Where was the data collected? e.g. home, clinic, workplace	**Page 6-7**
15. Presence of non-participants	Was anyone else present besides the participants and researchers?	**Page 6-7**
16. Description of sample	What are the important characteristics of the sample? e.g. demographic data, date	**Page 6-7**
Data collection		
17. Interview guide	Were questions, prompts, guides provided by the authors? Was it pilot tested?	**Page 7**
18. Repeat interviews	Were repeat inter views carried out? If yes, how many?	**Page 7**
19. Audio/visual recording	Did the research use audio or visual recording to collect the data?	**Page 7**
20. Field notes	Were field notes made during and/or after the interview or focus group?	**Page 7**
21. Duration	What was the duration of the inter views or focus group?	**Page 7**
22. Data saturation	Was data saturation discussed?	**Page 7**
23. Transcripts returned	Were transcripts returned to participants for comment and/or correction?	**NA**
**Domain 3: Analysis and findings**		

Data analysis		
24. Number of data coders	How many data coders coded the data?	**Page 8**
25. Description of the coding tree	Did authors provide a description of the coding tree?	**Page 8**
26. Derivation of themes	Were themes identified in advance or derived from the data?	**Page 8**
27. Software	What software, if applicable, was used to manage the data?	**Yes/ Page 7**
28. Participant checking	Did participants provide feedback on the findings?	**Page 7-8**
Reporting		
29. Quotations presented	Were participant quotations presented to illustrate the themes/findings? Was each quotation identified? e.g., participant number	**Page 8-18**
30. Data and findings consistent	Was there consistency between the data presented and the findings?	**Yes**
31. Clarity of major themes	Were major themes clearly presented in the findings?	**Yes**
32. Clarity of minor themes	Is there a description of diverse cases or discussion of minor themes?	**Yes**
