# Supplementary material for: Understanding the diabetes self-care behaviour in rural areas: Perspective of patients with type 2 diabetes mellitus and healthcare professionals
Source: PLoS One. 2024 Feb 8;19(2):e0297132. doi: 10.1371/journal.pone.0297132 (PMC10852243; doi:10.1371/journal.pone.0297132)
Supplement: S2 Appendix — (DOCX) [file pone.0297132.s002.docx]

**S2 Appendix**

**Supplementary Table 1: Number of FGDs in different sociodemographic subgroups (total FGDs=8)**

| **FGD subgroups** | **Total FGDs of subgroups** |
| --- | --- |
| **Gender** |  |
| Male | 4 |
| Female | 4 |
| **Age in year** |  |
| 30-60 | 6 |
| >60 | 2 |
| **Income (INR)/Month** |  |
| ≤ 40K (LIG) | 2 |
| 40-60K (MIG) | 3 |
| ≥60K (HIG) | 3 |
| **Education** |  |
| Illiterate | 4 |
| Literate | 4 |

**Abbreviation:** FGD- Focus Group discussions; LIG- Lower income group; HIG- Higher income group; MIG- middle-income group; INR-Indian rupee
