# Supplementary material for: Understanding the diabetes self-care behaviour in rural areas: Perspective of patients with type 2 diabetes mellitus and healthcare professionals
Source: PLoS One. 2024 Feb 8;19(2):e0297132. doi: 10.1371/journal.pone.0297132 (PMC10852243; doi:10.1371/journal.pone.0297132)
Supplement: S3 Appendix — (DOCX) [file pone.0297132.s003.docx]

**S3 Appendix:**

**Focus Group Discussion (FGD) Guide**

**Perception about diabetes and its management**

Let’s talk about your diabetes. **(Knowledge perception)**

**Probe:** How you were diagnosed with diabetes, emotional reaction to the diagnosis, and concerns. family response and their concerns? Is there something different or special in terms of the culture or the family? home remedies, diet, special foods.

**Perception on routine diabetes care**

According to you, are you doing enough to keep your blood sugar level under control? **(Perception on knowledge of routine diabetes care)**

**Probe:** How do you take care of your diabetes? (Diet, physical activity, blood glucose monitoring, medication adherence) What are you doing in your daily routine activity to manage your sugar? Do you understand how to follow your medication? How do you get your blood glucose level checked? What blood sugar level has your doctor suggested is good for you? What do you do when you feel shaky, hungry, and sweaty or when you feel thirsty, tired, and weak, or what do you do when you don’t feel well? What food choices would make a difference in your blood sugar control? What sort of physical activity is recommended to you by your physician?

Are you able to do as much as you wish to or required? Is it necessary to control blood sugar and do self-care activities? What makes it hard or challenging for you to take care of your diabetes? **(Perception on routine diabetes care challenges)**

**Probe: -**

1. **About medication adherence**

**Probe:**

1. Do you worry about side effects?
2. No reason to continue adhering to the treatment plan due to fatalism
3. Regular exercise and strict dietary precautions replace medication requirement
4. Busy
5. Forgetful
6. Lack of skills
7. No attempts were made to rectify the behaviour
8. Tired of taking medicines
9. Feeling deprived

**ii About a healthy diet**

What food choices would make a difference in your blood sugar control? What makes you feel difficult in adopting a diet plan?

**Probe:**

1. Favourite foods
2. Job nature
3. Family get together / social traditions/affordability

**iii About physical activity**

What sort of physical activity is recommended to you by your physician? Are you following it?

**Probe:**

1. Regularity
2. Confusing their daily work exertion as a replacement for regular exercise
3. Influence of weather

**iv What about foot care and stress management?**

What makes you decide when you should go to visit your doctor for a follow-up? **(Perception on health care needs for diabetes care)**

**Probe**: What types of health problems make you visit clinics or health centers? How often do you visit your doctor for regular check-ups? Do you able to understand the advice given by the doctor to manage diabetes?

**Suggestive measure for changes on diabetes care behaviour**

What is your opinion on present information and education about diabetes self-care?

**Probe:** What do you think about information available on diabetes self-care? What resources do you use to learn more about diabetes? Difficulties if any in comprehending the information?

How would you want to receive diabetes self-care information? How should you be trained and motivated to take better care of yourself?

**Probe:** Is there something missing that would be helpful to you to keep you better informed? If you need some support and I am ready to provide that support, what type of help would you require from me? What would help you stick to your treatment plan? For how long would you require my help?

**Closing** Thanks for coming. Your comments will be very helpful to me and the intervention

we are planning for all of you.

**In-depth Interview guide for Medical Officers**

Please share your experience in managing people living with diabetes?

Probe: About duration of working experience, how you involve yourself in managing people living with diabetes?

How do you provide facilities in the clinic to people living with diabetes? And How do you monitor the health progress of the patients and support them in their self-care issues?

Probe: How do you support the patients in the clinic? Particularly in guiding the patients for self-managing their diabetes.

What are the strengths of the current health services for people living with Diabetes?

Probe: How effective you find the process and in what way?

How much accessible do you think current health services for people living with diabetes?

Probe: OPD registration, Availability of medicine, Availability of health care professional, Availability of essential health care services.

What would you like to have to improve self-care practices in programme of non-communicable disease?

**Closing**

We have come to the end of our discussion.

Is there anything else you would like to add on the topics we have discussed today?

Thanks for coming. Your comments will be very helpful to me and the intervention

we are planning for People Living with Diabetes.

**In-depth Interview guide for ANM**

When you hear diabetes, what comes first in your mind? Will you explain to me?

Probe: What is symptoms and sign of diabetes? What is it? What is the consequences of diabetes?

Can this disease be prevented from happening?

Probe: - What is the reason for diabetes?

What do you think about information available on Diabetes?

Probe: Sources of information? Is there something missing that would be helpful to you to keep you better informed? How would you like to get that information?

How much accessible do you think current health services for people living with diabetes?

Probe: OPD registration, Availability of medicine, Availability of health care professional, Availability of essential health care services.

What are the things that patients consider before they decide whether or not to start diabetes management?

Probe: Different factor that affects the decision of treatment in people living with diabetes.

**Closing**

We have come to the end of our discussion.

Is there anything else you would like to add on the topics we have discussed today?

Thanks for coming. Your comments will be very helpful to me and the intervention

we are planning for people living with diabetes.
